# Management of infections for patient treated with ibrutinib in clinical practice

**DOI:** 10.3389/fonc.2024.1428464

**Published:** 2024-09-10

**Authors:** Claudia Baratè, Ilaria Scortechini, Sara Ciofini, Paola Picardi, Ilaria Angeletti, Federica Loscocco, Alessandro Sanna, Alessandro Isidori, Paolo Sportoletti

**Affiliations:** ^1^ Oncology Department, Hematology and Bone Marrow Transplant Unit, Pisa, Italy; ^2^ Clinic of Hematology, Azienda Ospedaliero Universitaria delle Marche, Ancona, Italy; ^3^ Department of Cell Therapies, Hematology Unit, Senese Hospital and University, Siena, Italy; ^4^ Hematology and Cellular Therapy, Mazzoni Hospital, Ascoli Piceno, Italy; ^5^ Onco-hematology, Terni Hospital, Terni, Italy; ^6^ Hematology and Stem Cell Transplant Center, Azienda Sanitaria Territoriale (AST) Pesaro and Urbino, Pesaro, Italy; ^7^ Hematology Unit, Azienda Ospedaliera Universitaria (AOU) Careggi, Florence, Italy; ^8^ Department of Medicine and Surgery, Institute of Hematology and Center for Hemato-Oncology Research (CREO), University of Perugia, Santa Maria della Misericordia Hospital, Perugia, Italy

**Keywords:** CLL, ibrutinib, infection, recommendation, prophylaxis

## Abstract

Ibrutinib, a highly effective inhibitor of the Bruton tyrosine kinase, has significantly transformed the therapeutic approach in chronic lymphocytic leukemia (CLL). Despite these advancements, the disease continues to be characterized by immune dysfunction and increased susceptibility to infections, with mortality rates from infections showing no significant improvement over the past few decades. Therefore, timely prevention, recognition, and treatment of infections remains an important aspect of the standard management of a patient with CLL. A panel of hematologists with expertise in CLL met to discuss existing literature and clinical insights for the management of infectious in CLL undergoing ibrutinib treatment. Despite not being a fully comprehensive review on the topic, this work provides a set of practical recommendations that can serve as a guide to healthcare professionals who manage these patients in their daily clinical practice.

## 1 Introduction

Chronic Lymphocytic Leukemia (CLL) is characterized by profound immune defects leading to severe infectious complications (1, 2). Immune dysfunction affects both innate and adaptive immunity, and both humoral and cell-mediated pathways. Hypogammaglobulinemia is the most common manifestation of immune deficiency that is present in approximately 85% of patients with CLL (IgG, IgA, and less commonly IgM deficiency) (3, 4). T cells are responsible for immunological dysfunction and immune complications (5) in both early and advanced stages. Patients have generally increased numbers of CD4+, CD8+ and regulatory T cells (Tregs), but are unable to optimally respond because of cells dysfunction and exhaustion. Furthermore, dendritic cells show incomplete maturation (6), and NK cell activation is also suppressed (1, 2).

**Figure 1:**
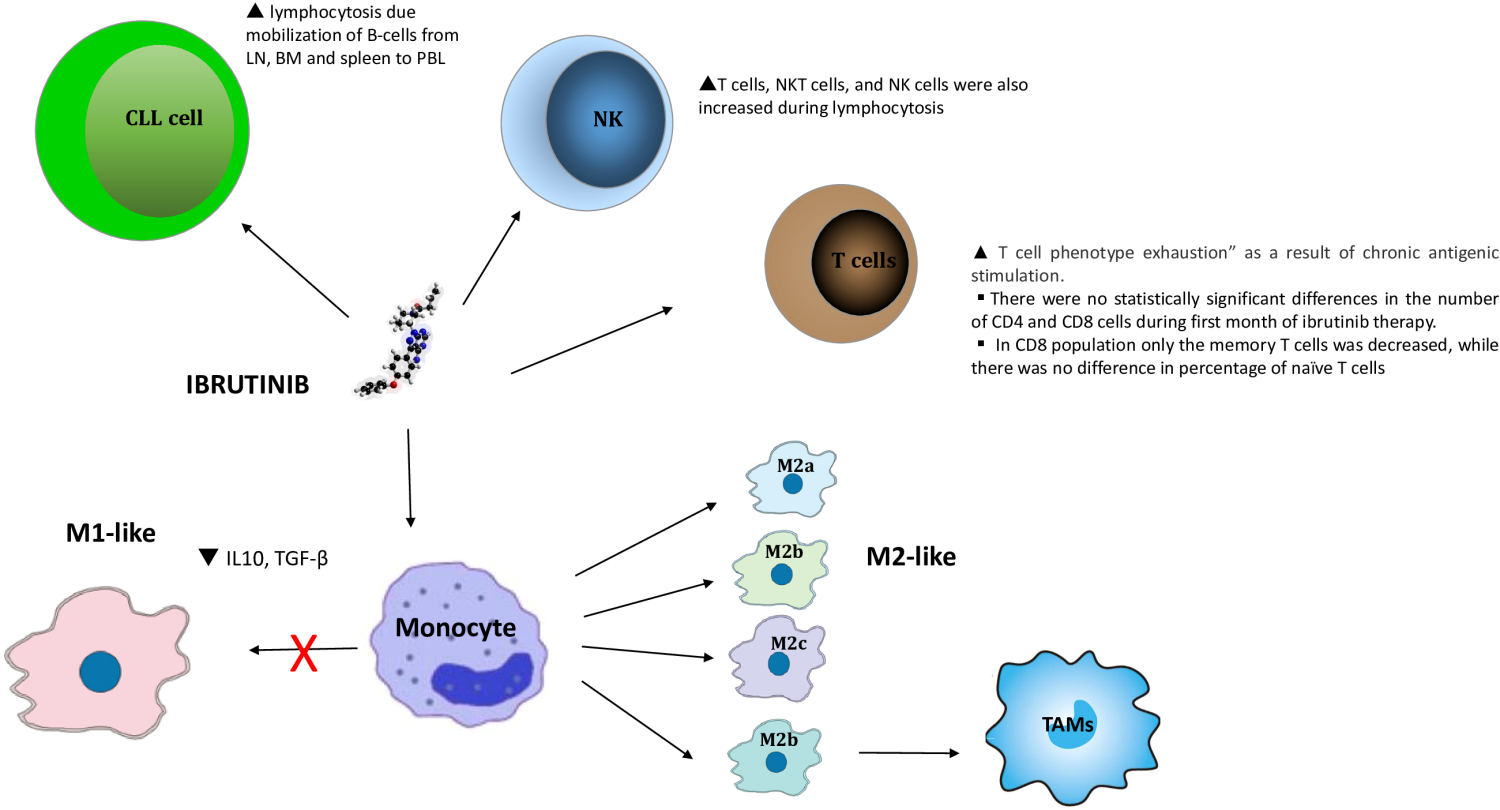
Chronic lymphocytic leukemia cells depend on interactions with the microenvironment. BTK inhibitors produce transient lymphocytosis caused by the mobilization of B-cells from LN, bone marrow and spleen to peripheral blood. They also produce changes in the tumor environment due to a decrease in the expression of immunosuppressive molecules such as PD-L1, IL-10, CD200 or BTLA in CLL B-cells. The heterogeneity and characterizations of macrophages. Macrophages could be roughly divided into two subtypes (M1-like and M2-like, while M2-like macrophages can be further differentiated into M2a, M2b, M2c, and M2d phenotypes.) depending on their different microenvironmental stimuli. All of these phenotypes express different cytokines, chemokines, and receptors which give rise to their different functions respectively. Generally, M1-like macrophages mainly induce proinflammatory responses and usually associated with Th1 response while M2-like macrophages contribute trophism and tissue tolerance. Furthermore, M2a is mainly mediating tissue repair and remodeling and Th2 responses; M2b is commonly responsible for immunoregulation; M2c mainly functions in phagocytosis, and M2d participates in angiogenesis in tumor. MC2 TYPE MACROPHAGE: M2c is stimulated by Il-10 and discharges CCL 16 and CCL 18 chemokines. The concentration of this cytokine was statistically significant lower at day 14 and at day 30 in comparison to pre-treatment concentration (day 0).

The intrinsic immune deficit in CLL is exacerbated by cytotoxic therapies, that also affect normal cells of the immune system (7). Infectious toxicity in patients with CLL treated with the Bruton tyrosine kinase inhibitor (BTKi) ibrutinib is most common in the first 6 months of therapy and is caused by on-target and off-target effects of this BTKi on NKs, CD4+ macrophages, CD8+ macrophages, Tregs and cytotoxic T lymphocytes (**Figure 1**). This effect decreases dramatically in the following months thanks to the partial restoration of the immune system by ibrutinib treatment (8–10). Ibrutinib’s irreversible inhibition of IL-2 inducible kinase (ITK) attenuates Th2 responses following T cell receptor (TCR) stimulation. This appears to enhance the anti-tumor immune response with an increased proportion of Th1 cells and decreased Treg cells, during the first six months of treatment (5, 11, 12).

In relapsed/refractory (R/R) patients with CLL, ibrutinib therapy results in an increased risk of infection in the early stages of treatment, which is reduced after a few months due to the improved immune response associated with the aforementioned effects on lymphocytes and immune system cells. The rate of Grade ≥3 infections in the first 6 months is 45% in patients with CLL treated with ibrutinib as second-line after chemotherapy (13) (including 21% pneumonias), whereas after 6 months the risk of infection is dramatically reduced. In patients aged 65 or older receiving front line Ibrutinib, the infection rate is lower, around 16% (14). The risk of infection is higher in R/R patients treated with ibrutinib after chemo-immunotherapy (CIT), due to the persistence of the immunosuppressive effect of CIT and the limited ability of ibrutinib to improve the intrinsic and therapy-related immunodeficiency of patients with CLL (15). Data from clinical trials were recently confirmed in a large Italian real-life cohort, where severe infections were approximately 18% in patients treated with first line BTKis, and 40% after CIT (16).

With the aim of addressing current challenges associated with infections during ibrutinib treatment for CLL, a team of experienced hematologists conducted this study, which presents our perspectives and offers practical recommendations to enhance patient care and outcomes.

## 2 Infectious risk assessment at diagnosis

The process of assessing infectious risk commences with obtaining a detailed medical history and anamnesis regarding prior infections. It is imperative to explore the patient’s potential history of previous infections or colonization, as well as individual predisposing factors such as treatment regimens, concurrent medical conditions, environmental exposures, occupation, presence of neutropenia, hypogammaglobulinemia, chronic obstructive pulmonary disease, and a history of tuberculosis. This information aids in guiding screening procedures, facilitating early detection, and determining appropriate empirical treatment strategies.

Screening for hepatitis B (HBV), hepatitis C (HCV) and human immunodeficiency virus (HIV) is mandatory before starting any kind of treatment for patients with CLL, including ibrutinib. Screening for hepatitis E virus (HEV) is discretionary and should be considered for patients displaying indicative signs and symptoms. The current recommendations (17) suggest assessing the serological status for HBV by testing for HBsAg, HBsAb, HBcAb, and, if HBsAg and/or HbcAb are positive, detecting HBV-DNA. For HCV, testing for anti-HCV antibodies is advised, with further testing for HCV-RNA if the antibodies are positive. Similarly, for HIV, testing for anti-HIV antibodies is recommended, with additional testing for HIV-RNA if the antibodies are positive. If any of these markers are positive, the patient should be referred to an infectious disease specialist before initiating antiviral treatment. It is noted that active HBV infection, indicated by HBV-DNA positivity or HBsAg positivity, does not preclude the use of Ibrutinib therapy as long as appropriate antiviral treatment is administered concurrently, with close viral monitoring overseen by an infectious disease specialist (18, 19).

Epstein-Barr virus (EBV) serology testing is not recommended for routine screening purposes, but should be considered in cases where there are elevated transaminase levels of grade II or higher. Monitoring for Cytomegalovirus (CMV) in patients with serology indicating prior exposure is advised only for those at high risk of CMV reactivation who are undergoing treatment with a combination of Ibrutinib and anti-CD20 antibody. It is not recommended to conduct serological screening for CMV in patients initiating Ibrutinib therapy alone (17–19).

Quantiferon or tuberculin skin testing (TST), or a combination of the two tests, to detect latent tuberculosis infection is recommended in high-risk patients before starting Ibrutinib (20). Patients who previously had active tuberculosis that was properly treated do not need to be screened or receive preventive treatment. For patients with previous active tuberculosis that was not adequately treated, seek expert advice and consider full tuberculosis treatment or preventive treatment, depending on the history of tuberculosis (21).

Finally, it is important to measure immunoglobulin values, in particular IgG, before starting treatment with Ibrutinib, in order to adequately correct hypogammaglobulinemia and thus reduce the risk of infections (see the specific chapter of this manuscript for further details).

## 3 Anti-infective prophylaxis

CLL is well-known for increasing susceptibility to infections due to both cell mediated and humoral immunity impairment, as well as complement deficiency (1, 2). When Ibrutinib is administered, the incidence of severe neutropenia (grade ≥ 3) in treatment-naïve (TN) patients is 10%, while the occurrence of severe pneumonia (grade ≥ 3) is 4%, and severe infections (grade ≥ 3) are observed in 23% of cases (22). When Ibrutinib is used in combination with an anti-CD20 monoclonal antibody, the rate of severe pneumonia (grade ≥ 3) is 7%, and the incidence of severe neutropenia rises to 36% (23). In R/R patients, severe neutropenia (grade ≥ 3) is reported in 16% of cases, with severe pneumonia (grade ≥ 3) occurring in 7% of patients, and severe infections (grade ≥ 3) observed in 30% of cases (24). Pneumonia continues to be the most common infectious complication in CLL, with an incidence of 12% based on an integrated analysis of landmark ibrutinib studies (25). Currently, the use of antibacterial prophylaxis during ibrutinib therapy is not standard practice and is not advised during ibrutinib treatment due to the minimal risk of mucosal injury. Based on those data, patients with CLL on Ibrutinib treatment, should be considered at intermediate risk for pneumonia and fluoroquinolone prophylaxis should be considered during neutropenia (or trimethoprim/sulfamethoxazole in intolerant). When fluorquinolone is indicated, side effects must be monitored due to risk of prolonged QT interval; moreover, ciprofloxacin inhibits hepatic cytochrome P450 isoenzyme 1A2, which can impair elimination of substrate drugs (26, 27).

Pneumocystis jirovecii (PJP) prophylaxis is not recommended as a standard practice, primarily due to the relatively low occurrence rate of PJP infection under ibrutinib (28–30). However, it may be advisable to consider PJP prophylaxis within the initial six months of therapy for individuals who have previously undergone treatment with alemtuzumab, purine analogues, or high doses of steroids (e.g., 20 mg daily for a minimum of three weeks) and have a history of prior PJP infection, or those at elevated risk due to underlying conditions such as diabetes, chronic pulmonary or renal disease. The use of anti-CD20 antibodies in prior therapy does not appear to significantly impact the risk of infection. Trimethoprim-sulfamethoxazole has been shown to be effective for PJP prophylaxis, typically administered at a daily dosage of 80/400 mg or 160/800 mg three times weekly (31). In cases of allergic reactions, Pentamidine aerosol may be a preferable alternative for prophylactic treatment (27).

Antifungal prophylaxis is not generally recommended. Incidence of invasive fungal infections in real-life studies is low (about 2-3%) and Aspergillus is the most frequent etiology. Secondary antifungal prophylaxis for a previous invasive fungal infection (IFI) must be considered. Active surveillance is highly recommended for high-risk patients, as defined by prolonged neutropenia duration, prolonged steroids treatment or 2 or more lines of previous therapy (18, 32, 33).

Antiviral prophylaxis is typically not advised, particularly for individuals who have already been vaccinated with recombinant zoster vaccine (RZV). However, in specific cases where patients have not previously received RZV, have experienced reactivation of varicella-zoster virus within the past 12 months prior to initiating therapy, or are prone to recurrent HSV infections, it may be appropriate to consider prophylactic treatment with clat at daily dosage of 400 milligrams BID during the initial 6 months of ibrutinib therapy (26, 34).

CMV reactivation could be monitored in seropositive patients at high risk. Pre-emptive therapy is not a standard of care at first reactivation, but should be considered in case viral load is rising, even if a cut off value is not available in this setting of patients. Ganciclovir 5 mg/kg BID could be used, monitoring the risk of bone marrow suppression (19, 26).

Reactivation of HBV infection is a well-documented complication observed in individuals with hematological malignancies undergoing immunosuppressive treatments such as chemotherapy, monoclonal antibodies (e.g., antiCD20 antibody), or stem cell transplantation. The likelihood of reactivation varies among patients with chronic HBV infection, characterized by the presence of hepatitis B surface antigen (HBsAg+), and those with prior exposure to HBV, identified by the presence of antibody to hepatitis B core antigen (anti-HBc+). For patients positive for HBsAg+, it is advisable to assess viral load and recommend antiviral therapy for those with detectable HBV-DNA levels, similar to the approach taken for the general population. As mentioned in a previous section, it is advised to conduct HBV screening in patients with CLL who are undergoing immunosuppressive or cytotoxic treatment. Antiviral prophylaxis is recommended for a period of 12-18 months following the completion of immunosuppressive therapy. Entecavir 0.5 mg daily is our preferred antiviral medication for prophylaxis due to its low risk of resistance development. Tenofovir, 300 mg daily (TDF) or 25 mg daily (TAF) can be also considered as a valid option that requires monitoring of renal function due to the potential nephrotoxicity (26).

However, in regions where cost and availability may vary, lamivudine could also be considered, especially for patients with an undetectable viral load (26, 35, 36).

Prophylaxis of HBV reactivation in patients with prior HBV exposure (HBsAg-/anti-HBc+) still remains a matter of debate. However, it is usually adopted, in particular for patients treated with an anti-CD20 monoclonal antibody or a stem cell transplant, and should be continued for at least 12 months after the end of therapy. During HBV prophylaxis, it is mandatory to monitor HBV-DNA and liver function at least every 3-6 months. Detection of anti-HBs positivity in a patient who is anti-HBc+ and HbsAg –, prophylaxis has been suggested as a protective factor against HBV reactivation, but it is not clear which titer of antibodies should be used to guide or avoid prophylaxis.

Ibrutinib is a relatively new agent for CLL treatment. Patients with previous or chronic HBV infection were excluded from clinical trials, so it remains unclear whether ibrutinib treatment is associated with an increased risk of HBV reactivation in patients with previous infection. The summary of product characteristics of ibrutinib states that HBV reactivation may occur during treatment and recommends serological testing for HBV and HCV prior to starting ibrutinib treatment (27). In real-world reports of patients treated with ibrutinib, HBV reactivation is described, both in patients who are HBsAg+ and HbsAg-/anti-HBc+, with a remarkable variability in number and severity of reactivation (37–41). Among others, the GIMEMA retrospective experience did not report a significative rate of reactivation in two cohorts of HBsAg-/antiHBc+ patients treated with Ibrutinib with or without lamivudine prophylaxis. The cumulative incidence of reactivation was 1.9% (42). Whereas in patients treated with tyrosine kinase inhibitors like imatinib and nilotinib the American Gastroenterology Association (AGA) indicates a moderate risk for HBV reactivation, no suggestions are currently given for patients receiving ibrutinib and other BTKi (43). The specific mechanism underlying HBV reactivation in individuals undergoing Ibrutinib therapy remains uncertain, thus raising questions regarding the optimal approach between prophylaxis and monitoring with pre-emptive therapy. The guidelines outlined by the European Conference on Infection in Leukemia (ECIL-5) do not recommend prophylactic measures for HBV seropositive patients undergoing ibrutinib monotherapy (44). In individuals who are HbsAg+, prophylactic measures may be considered due to the infrequent occurrence of HBV reactivation and the extended duration of Ibrutinib therapy. However, there is no consensus regarding the necessity of prophylaxis versus monitoring for those who are HBcAb+/HbsAg-. It is essential to seek guidance from an infectious disease specialist and adopt a multidisciplinary approach to monitor patients closely. This approach enables the prompt initiation of preemptive therapy in the event of a concerning elevation in liver transaminases, whether related to HbsAg or HBV DNA.

## 4 Vaccinations and immune responses

Since the infectious risk has been mostly associated with immunosuppression in patients with CLL, it is recommended to plan a vaccination strategy at diagnosis, independently from treatment initiation. It is important to keep in mind that the immunological response, intended as an antibody response to vaccination, is partially compromised, in part due to the disease itself, and by the treatments we adopt, in particular if we use chemotherapy or monoclonal antibodies. Anti-pneumococcal, anti-meningococcal, anti-seasonal flu, and anti-VZV vaccines are strongly recommended. A variable response to different types of vaccines has been described in different studies. Two clinical trials evaluating the recombinant vaccine for HBV (HepB-CpG) and for herpes zoster virus, report a different response based on the status of the disease and the type of vaccine itself. The rate of response to HepB-CpG adjuvant was found to be lower among patients receiving ibrutinib therapy and other BTKis at 3.8%, compared to treatment-naive patients at 28.1%. Conversely, the response rate to RZV did not show a significant difference between the BTKi group (41.5%) and the treatment-naive group (59.1%).

The antibody response to RZV was long-lasting and remained stable for at least two years after vaccination. No significant differences in cellular and humoral responses were reported between ibrutinib and acalabrutinib; nor differences in vaccine response in patients who underwent ibrutinib as first line compared to subsequent lines. Finally, no correlation was observed between cellular or antibody response and age or immunoglobulin level (45). Interestingly, approximately 39% of patients mount a T cell response despite having a negative antibody response to RZV (46). Individuals with CLL are at a higher risk of developing severe respiratory syncytial virus (RSV), which can necessitate hospitalization. Vaccination against RSV would therefore be recommended, even though it is currently only approved for people who are 60 years of age or older. In this respect, the RENOIR trial documented how preventing RSV is associated with lower respiratory tract illness and RSV-associated acute respiratory illness in adults ≥60 years with a good safety profile (47). In general, a worse antibody response was also observed for anti-SarsCov2 vaccination in patients with CLL, generally in those receiving treatment with ibrutinib or BCL2i in association with antiCD20 antibody. Antibody response to the pneumococcal vaccine (PVC13) in CLL patients was documented by an Italian study, which shows that only 8% of patients with CLL develop an antibody immune response, with no responses in patients treated with chemo-immunotherapy. Age, immunoglobulin levels, previous treatments and disease progression were associated with lower response (48). ​​Seasonal influenza vaccination in patients with CLL treated with ibrutinib was tested in a phase 2 trial of single-agent ibrutinib (NCT01500733) and documented that up to 74% of patients achieved seroprotective titers against viruses after vaccination with an acceptable safety profile (49). Tetanus vaccines are available in two forms: tetanus, diphtheriae (Td), which protects against tetanus and diphtheriae and tetanus, diphtheriae, and pertussis (Tdap) protect against all three bacteria. A Td or Tdap routinely should be given every ten years. Data on tetanus vaccine responses in CLL are sparse, and there are no data on responses to pertussis vaccines in patients with CLL. The degree of immunization improves if the disease is under control or in early stage disease (50).

Therefore, when possible, and after a careful evaluation of the risk/benefit ratio, we recommend a vaccination program that is as complete as possible, at the time of CLL diagnosis or right before the initiation of treatment. In fact, the response to vaccines up to 12 months after the end of treatment with antiCD20 antibody appears absent and therefore it would be better to bring vaccinations forward before this treatment or, if a patient is already on therapy, to defer vaccination. However, deferring vaccination during continuous therapy with ibrutinib is impossible, and thus we should vaccinate the patient when the disease is under control. Instead, some limitations regarding vaccinations concern the persistence of the vaccine response once treatment has begun. Vaccination history should be re-evaluated at the end of treatment, in order to plan an individualized vaccination program based on age, comorbidities and national recommendations. Measuring a specific antibody titer after vaccination would help to estimate an adequate response, however variability in effective immune response between individual subjects with CLL implies that cannot be translated into clinical practice (51).

## 5 Immunoglobulin therapy

Approximately 85% of patients with CLL have hypogammaglobulinemia, which may or may not be associated with recurrent infections. The current ESMO and NCCN guidelines state that prophylaxis of bacterial infections with immunoglobulins is not routinely recommended because no beneficial effect on overall survival has been demonstrated (26, 52), moreover this is an important strategy for patients with recurrent sinopulmonary infections requiring intravenous antibiotics or hospitalization (26). Given these indications for each patient, it therefore appears critical to perform endogenous immunoglobulin dosing and infectious event monitoring to identify those who may benefit from exogenous immunoglobulin administration. Immunoglobulin supplementation is commonly administered through intravenous (IV) or subcutaneous (SC) routes. IV products are typically administered at a dosage of 0.3-0.5 g/kg every 3-4 weeks, with the option to adjust the dose and frequency based on factors such as Ig levels, frequency of infections, and individual patient requirements (26, 53). Similarly, the dosage and frequency of SC products may also vary.

Only patients with CLL and severe immunoglobulin deficiency, defined by serological IgG levels< 400-500 mg/dl (26, 52) and recurrent (>3 episodes) or severe (> grade 3-4 infection episode) bacterial infections may receive immunoglobulin supplementation, in order to maintain IgG dosages around 300-500 mg/dl (26, 52). The major limitation to the use of immunoglobulins depends on their difficulties in procurement, especially in the post Covid19 era, due to the shortage of donors. A specific issue for SC administration is the availability of pumps for home self-infusion, which are in short supply and often require patients to travel to their hospital referral center for infusion. Recent research conducted on patients with secondary hypogammaglobulinemia has revealed no significant disparities in terms of infectious occurrences or mortality rates between two distinct formulations of immunoglobulin, whether administered SC or IV. Some recent publications tried to compare efficacy and tolerability of the two formulations of Ig in patients with hematological malignancies in general and with B cell neoplasms in particular. Spadaro at al. reported the cases of 14 patients with B cell malignancies and secondary hypogammaglobulinemia, and compared the treatment with IVIg and SCIg. They did not observe any differences in terms of infections, adverse events but only an higher value of Ig levels with SC form (54). Another small study enrolled 30 patients with hematological malignancies, most of them in complete remission, and compare SC and IV formulations of Ig in order to identify differences in term of infection incidence, hospitalization but also patient preferences. Again, author did not find so many differences in the two subgroups of patients, but they reported a good handling, ease of use and patient preference for SCIg (55). Even with the small number and heterogeneity of diagnosis of patients enrolled in these studies and the limits of a retrospective analysis, these data are interesting in order to improve the use of SCIg in our patients with CLL.

Focus on patients with CLL especially treated with ibrutinib was recently published a single center retrospective analysis which included 27 patients with relapsed/refractory (R/R) CLL received ibrutinib monotherapy at least for one year. Intravenous immunoglobulin (IVIg) treatment was initiated in 9/27 patients with frequent infections and IgG levels below 500 mg/dL. They demonstrated that OS and PFS were almost the same for patients with or without secondary hypogammaglobulinemia, but the second group had a higher incidence of adverse event, especially pneumonia which cause a temporally or permanent reduce of ibrutinib dose they also suggest to start IVIg replacement when IgG level is below 650 mg/dl and recurrent infection (56).

A larger retrospective analysis of 86 hematology centers in Germany, collected from 1086 patients (CLL 490, MM 596) in order to analyzed the guidelines adherence (GLAD) and rates of hospitalization and infections. Of all patients with CLL, 115 (23.5%) received IVIg replacement. Patients with higher GLAD score had a lower incidence of infections and severe complications. In the multivariate analysis other risk factors for infections events are higher Charlson comorbidity index, existing hypogammaglobulinemia below 4 g/l and advanced line of therapy (57).

The optimal duration of immunoglobulin therapy remains a topic of debate within the medical community. Clinical guidelines advocate for an individualized approach tailored to each patient. The determination of whether to maintain or discontinue immunoglobulin therapy, as well as the frequency of administration, should be guided not solely by serum immunoglobulin levels, but primarily by the occurrence of significant infections. Consequently, the frequency of immunoglobulin administrations may be decreased if a patient remains free of infectious episodes for an extended period, with a return to monthly administrations warranted in the event of new infections.

## 6 Infectious disease workup

Novel therapies, especially small molecules, have proven to be effective and have a favorable toxicity profile, but infections continue to represent a significant complication also in the era of novel agents.

Upper respiratory infections and urinary infection were the most frequent infectious complications occurring during Ibrutinib treatment. Penumonia occurred in 13% during Ibrutinib treatment and 8% during Acalabrutinib (58).

As concern fungal infections, they generally occurred in the first 6 months of treatment with ibrutinib. Invasive fungal infection was rare in patients taking ibrutinib, affecting < 1.2% of patients: aspergillus species were the most frequent agent involved (61% of fungal infections) followed by Cryptococcus species (25%) (32, 59, 60).

Based on our clinical expertise and the above-mentioned evidences, we suggest the following infectious workup in patients with signs or symptoms of infection during therapy with ibrutinib:

- Blood cell count, chemistry for evaluation of organ dysfunction, C-reactive protein level, measurement of biomarkers of sepsis such as procalcitonin levels;
- Blood culture; urine analysis and culture; sputum culture test;
- Chest radiology and chest CT scan in order to identify a specific infection site and evaluate a specific pattern (interstitial pneumonia vs other pattern);
- Serologic test to identify atypical pneumonia (*Mycoplasma pneumoniae, Chlamydia, Coxiella Burnetii*); urinary antigen tests for detection of Legionella and Pneumococcal antigens; PCR viral respiratory panel;
- Abdominal ultrasonography, CT san and MRI only based on clinical presentation (if biliary or non-bliary abdominal source of infection is suspected);
- Galactomannan and β-d-glucan if fungal infection is suspected;
- The search for herpetic viruses, mycobacteria and cryptococcus should be performed based on clinical suspicion.

If first-level investigations are not diagnostic, we generally implement with targeted examinations at the site of infection using:

- Bronchoalveolar lavage for culture test;
- Histopathological examination of tissue biopsies in order to identifying infectious pathogens directly from tissue biopsies by microscopic visualization;
- Lumbar Puncture and Central Nervous System RMN if neurological signs or syntoms are present (in order to identify fungal and/or viral infections).

## 7 Drug temporary interruption during infections

The biggest dilemma in a febrile patient with CLL under control in the outpatient setting is whether or not temporarily interrupt the treatment and, if yes, for how long. In our clinical practice, if a patient presents with new-onset fever and has been hospitalized due to fever, we prefer to temporarily interrupt ibrutinib until a diagnosis is made, and the patient shows significant improvement. In these patients, it is not always clear to us whether the drug can be restarted, as resumption of ibrutinib may facilitate the development of recurrent inflammatory pneumonia in some cases. However, in the majority of patients with typical bacterial or viral pneumonia, ibrutinib can be resumed once the patient has recovered. Patients developing IFI during ibrutinib therapy should be carefully discussed with the infectious disease specialist, in order to establish if and when ibrutinib should be resumed. Furthermore, in patients with suspected or confirmed IFI, it is important to remember that ibrutinib has a significant interaction with both voriconazole and posaconazole. Alternative therapy with isavuconazole has fewer significant drug interactions, but this drug has been less widely used. In these patients, ibrutinib often needs to be discontinued for an extended period of time to achieve complete control of the infection (4, 48, 61–63).

In patients who experience a new-onset fever, which can be controlled within 72 hours with or without empirical antibacterial therapy at home, ibrutinib should be continued, and eventually discontinued if fever or symptoms of infection worsen after 72 hours of observation or antibiotics therapy.

Our practical suggestion is to base the decision whether or not temporarily or permanently interrupt the drug on clinical experience, referring to what is reported in the summary of product characteristics which might be found at https://www.ema.europa.eu/en/documents/product-information/imbruvica-epar-product-information_en.pdf.

## 8 Current recommendations for SARS-COV-2

Patients with CLL are at increased risk of poor COVID-19 outcomes, compared with the general population. Since the early phases of COVID-19 pandemic, the management of CLL have been significantly reshaped and patient preferences about CLL therapy options have been influenced by the risks associated with SARS-CoV-2 infection (64).

Large cohort studies on patients with CLL reported 30-40% case fatality rates in early cohorts of severe COVID-19 (65–67) and a trend toward lower mortality over time as infection management improved (68, 69). The infection with the new SARS-CoV-2 variants has been described as a milder disease in the general population and in patients with hematological malignancies than the infection with the first variants (70). During the Omicron era, it was reported that CLL patients also had a lower risk of dying from COVID-19 (71). However, patients with CLL may have a higher risk of developing persistent COVID-19 infection with a higher risk of significant morbidity and mortality (70), especially if they received anti-CD20 monoclonal antibodies (72). If a patient has severe COVID-19 and requires hospitalization and oxygen, it is important to find out if treatment can be delayed until the patient is free of the infection. CLL therapy can be continued in the majority of mild infection cases.

The rapid development of vaccines against SARS-CoV-2 had a major role in decreasing COVID-19 related mortality and hospitalizations, both in clinical trials and in nationwide studies (73, 74). Therefore, vaccination remains the first line of prevention and experts have generally recommended that patients with CLL should be vaccinated as soon as possible (75). In CLL, the protective effects of vaccination against a variety of pathogens are variable, and the SARS-CoV-2 virus is not an exception. Numerous studies evaluated antibody response to COVID-19 vaccination in CLL and only few assessed T-cell–mediated responses. Patients with CLL displayed the lowest seropositivity rate (humoral response range of 40%-67%) compared to almost 100% in healthy subjects (76–79).

Being on active treatment has been reported as the most commonly independent factor for poor response with a median seroconversion rate of 52%, 28% and 17% for venetoclax monotherapy, BTKis (including ibrutinib) and antiCD20 antibody treatment, respectively (80). Notably, patients who received a vaccine at least a year after the end of anti-CD20 antibody therapy had a better response (81). Response rates for patients in remission were similar to those who were treatment-naïve. Antibody titers in vaccinated patients with prior COVID-19 infection were comparable with titers seen in healthy controls (36) indicating that the use of vaccine can increase a generally impaired humoral response of patients with CLL to SARS-Cov2 infection (76). Outside of a research study, antibody testing is not recommended to measure immunity after vaccination.

Besides antibody production, T cell responses were associated with an improved outcome in COVID-19 patients, suggesting the importance of SARS-CoV-2–specific cellular responses for protective immunity (82). Evidence supported that the administration of SARS-CoV-2 vaccines could elicit T-cell responses in the absence of antibody (78, 83), highlighting the possibility of a cell-mediate protection in patients with CLL and a poor serological conversion. Nevertheless, the assessment of cellular responses is challenging and not recommended in regular clinical practice.

No evidence exists to support stopping CLL treatment to get vaccinated. Whenever possible, it is best to get vaccinated before starting treatment that should not be delayed to complete the vaccine series anyway. As vaccination programs have accelerated, many patients with CLL have received a third dose and additional updated booster may provide improved protection against newer variants. Seropositivity rates in patients with CLL have been shown to rise after each vaccination, sometimes even when a prior immune response had not been present (84). Moreover, a booster vaccination schedule might allow to manage the decay in antibody response over time that has been described to be similar in patients with CLL and healthy controls aged ≥70 years (65). Based in these observations, third dose and booster shots are recommended in CLL (75).

The use of antiviral agents preventing viral replication represents an additional therapeutic strategy in the management of COVID-19. Given the potential for drug-drug interactions with the SARS-CoV-2 antiviral medications and the short course of the antiviral therapy, a precautionary wait on antileukemic therapy may be advised. The antiviral Remdesivir was approved for the treatment of COVID-19 based on clinical data demonstrating that this drug accelerates recovery in individuals hospitalized with lower-respiratory tract infection. In a recent retrospective analysis Tadmor et al (85) showed the beneficial effects of prompt therapy with the drugs nirmatrelvir and ritonavir for patients with CLL who test positive for SARS-CoV-2. This study demonstrated for the first time that patients with CLL receiving early antiviral treatment after a positive SARS-CoV-2 test can lower their risk of hospitalization and mortality. Benefits of such antiviral approach may be especially beneficial in patients with CLL who are over 65, are heavily pretreated, have comorbidity associated with increased risk of infections, or are receiving immunoglobulin treatment. There is a requirement for dose adjustment of ibrutinib under the treatment with these drugs.

In order to lower the risk of severe infection, we suggest to consider prophylactic anti-SARS-CoV-2 monoclonal antibody injection to patients with CLL on active treatment, including anti-CD20 antibody therapy. In a phase III trial, participants who received both tixagevimab and cilgavimab (Evusheld) had a decreased incidence of symptomatic and severe COVID-19 disease (86). Pharmacokinetic findings demonstrated that the monoclonal-antibody combination was persistent in the serum for 6 months following delivery. Based on these findings, the FDA authorized the use of tixagevimab plus cilgavimab as pre-exposure prophylaxis of COVID-19 in people who were not expected to mount an adequate immune response vaccination as patients with CLL (87) (FDA). However, as with vaccines, it will be important to follow changes over time in circulating variants that may evade protection, thereby limiting the efficacy of tixagevimab/cilgavimab. In a single institution study, patients with B-cell malignancies were shown to have breakthrough COVID-19 infections (11%) in the Omicron period despite getting tixagevimab and cilgavimab passive immunization and being fully vaccinated (88). Hospitalization rates were low and mortality was below 2% for patients with CLL, suggesting benefit from this strategy. However, the subgroup of CLL with comorbidity still had mortality above 20% (71). In a population-based study, patients with CLL over the age of 65 were the only subgroup of patients with excess mortality following COVID-19 infections in 2022 during the Omicron era (89).

Anti-SARS-CoV-2 monoclonal antibody injection is not a substitute for vaccination but may provide an additional layer of protection against symptomatic infection. It remains critical that CLL individuals keep up to date with COVID-19 vaccination, take precautions to avoid infection and be tested for SARS-CoV-2 infection if they experience signs and symptoms consistent with COVID-19 in order to be promptly seek for medical attention in case of active infection.

## 9 Practical points

### 9.1 Infectious risk assessment before ibrutinib treatment

#### 9.1.1 Comprehensive patient history

- Anamnesis and comorbidities.
- Investigating past infections/colonization and individual predispositions guides screening, early diagnosis, and empiric therapy.

#### 9.1.2 Viral screening before ibrutinib

- Screening for HBV, HCV, and HIV (before starting any kind of treatment for CLL).
- EBV serology testing is post-screening, triggered by increased transaminases.
- CMV monitoring is indicated in high-risk patients receiving ibrutinib and anti-CD20 antibody combination therapy, no serological screening for ibrutinib monotherapy.

#### 9.1.3 Tuberculosis screening

- Quantiferon testing or tuberculin skin testing is recommended in high-risk patients before Ibrutinib.
- Properly treated past tuberculosis cases may not require screening, however IDs consultation is essential for inadequately treated cases.

#### 9.1.4 Immunoglobulin assessment

- Measuring immunoglobulin values, especially IgG, pre-Ibrutinib, is crucial (**Table 1**).

**Table 1:** Infectious Risk Assessment.

		Time	
		Diagnosis	Before treatment Anytime
**Comprehensive Patient History**	**Anamnesis and comorbidities**	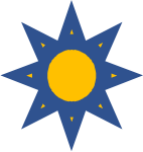 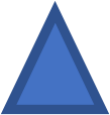	
	**Past infections/colonization**	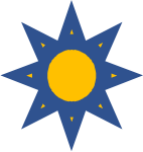 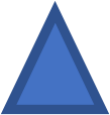	
**Viral Screening**	**HBV, HCV, and HIV**	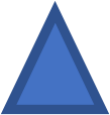	
	**EBV**	** 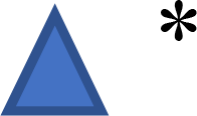 **	
	**CMV**	** 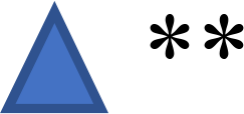 **	
**Tuberculosis**	**Quantiferon testing or tuberculin skin testing**	** 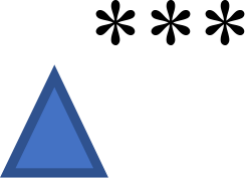 **	
**Immunoglobulin assessment**		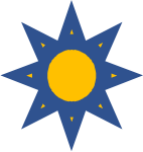 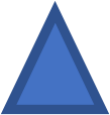	

*EBV serology testing is post-screening, triggered by increased transaminases.

**Indicated in high-risk patients receiving ibrutinib and anti-CD20 antibody combination therapy.

***Recommended in high-risk patients.

### 9.2 Anti-infective prophylaxis in patients with CLL on ibrutinib

#### 9.2.1 Antibacterial prophylaxis

- Routine anti-infective prophylaxis, including antibacterial agents, is not recommended due to the low risk of mucosal damage.

#### 9.2.2 Pneumocystis jirovecii prophylaxis

- Routine PJP prophylaxis is not recommended, in specific high-risk cases, including previous treatment with certain agents and comorbidities, it should be considered.

#### 9.3.3 Antifungal prophylaxis

- Antifungal prophylaxis is not generally recommended, owing to the low incidence of invasive fungal infections.

#### 9.3.4 Antiviral prophylaxis

- Routine antiviral prophylaxis is not generally recommended, especially for those previously vaccinated against herpes zoster.
- Selected patients with specific risk factors may be considered for aciclovir prophylaxis during the first 6 months of ibrutinib treatment.

#### 9.5.5 Hepatitis B virus reactivation and prophylaxis

- Prophylaxis for HBV reactivation is recommended in patients with chronic HBV infection (HBsAg+).
- The risk of reactivation in patients with prior HBV exposure (HBsAg-/anti-HBc+) is low, both prophylactic treatment with lamivudine and monitoring are accepted (**Table 2**).

**Table 2:** Anti-infective prophylaxis in patients with CLL on ibrutinib.

Prophylaxis	Recommended	
	YES	NO
Antibacterial		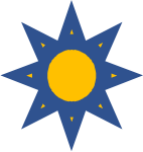
PJP		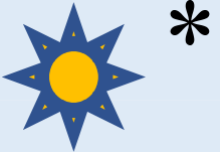
Antiviral		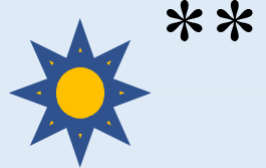
HBV ( HBsAg+)	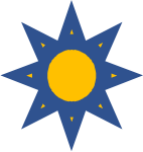	
HBV(HBsAg-/anti-HBc+)	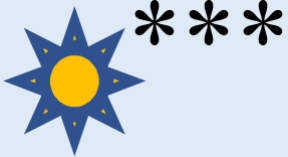	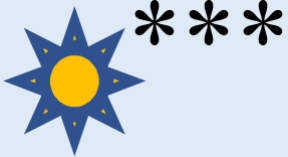
Antifungal		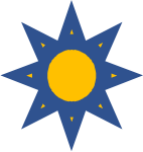

*In specific high-risk cases should be considered.

**Selected patients with specific risk factors may be considered for aciclovir prophylaxis during the first 6 months of ibrutinib treatment.

***Both prophylactic treatment and monitoring are accepted.

### 9.3 Vaccinations and immune responses

1. Clear recommendations emerge for pneumococcal, meningococcal, seasonal influenza, and VZV vaccinations.
2. Variability in responses to different vaccines highlights the complexity of the situation.
3. Timing of vaccinations is crucial, whether possible it should be administered before starting treatment (**Table 3**).

**Table 3:** Recommendation plan for a vaccination strategy.

Pathogen	Non-live vaccines types	MA	Dose series	Time to start		Duration Revaccinate	Sero Conversion %
				Before treatment*	Anytime**		
Pneumococcal	PPSV23	IM/SC	1	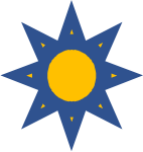 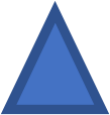		every 5 y	0-100 (90)
Haemophilus influenzae	Hib	IM	1	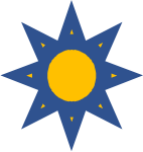 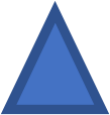		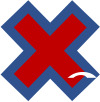	48 (91)
Respiratory syncytial virus	RSV	IM	1	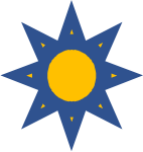 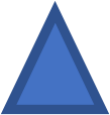		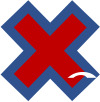	41 (92)
Meningococcal	MenACWY MenB	IM	2 2	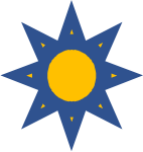 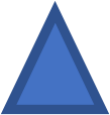		every 5 y	No data
Influenza	IIV4, ccIIV4, RIV4, aIIV4; HD-IIV	IM	1	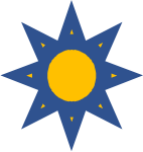 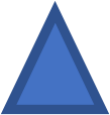		every year	0-42 (90)
Herpes Zoster	VZV	IM/SC	2	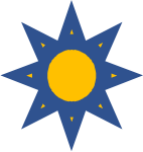 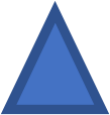		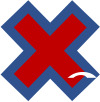	40-80 (90)
Sars Cov2	Covid19	IM	1	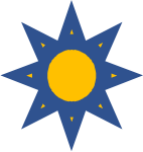 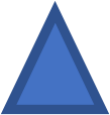		every year	18-60 (90)
Tetanus Diphtheria	Td	IM	1	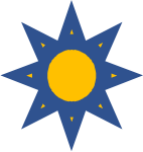 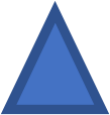		every 10 y	No data

*Better before antiCD20 antibody therapy. Defer after 12 months from the end of chemoimmuno-therapy.

**
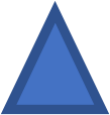
 During target therapies (BTKi or Bcl2i) prefer when disease is under control.

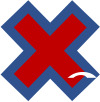
 No Guidance/ No evidence.

MA mode administration: IM intramuscularly; SC subcutaneously.

See Advisory Committee on Immunization Practices (www.cdc.gov/vaccines/acip) and Centers for Disease Control and Prevention (www.cdc.gov) and ASCO guideline (DOI https://doi.org/10.1200/JCO.24.00032).

### 9.4 Immunoglobulins and hypogammaglobulinemia

1. The majority of patients with CLL exhibit hypogammaglobulinemia.
2. Current guidelines do not recommend routine prophylaxis with immunoglobulins, however individual assessment is crucial.
3. Administration of immunoglobulins can vary between intravenous (iv) and subcutaneous (sc), with considerations on frequency and dosage.
4. The decision to continue or discontinue immunoglobulin treatment should be based on serum values and the presence of severe infections (**Table 4**).

**Table 4:** Immunoglobulins and Hypogammaglobulinemia.

Number pt	85% of CLL
Administration route	intravenous or subcutaneous
Dosage	0.3-0.5 g/kg every 3-4 weeks (IV)
Indication for therapy	IgG levels< 400-500 mg/dl and recurrent bacterial infections
Goal	300-500 mg/dl

### 9.5 Infectious workup during ibrutinib therapy

#### 9.5.1 Clinical presentation assessment

- Blood cell count, chemistry, C-reactive protein, and procalcitonin.
- Blood and urine cultures, sputum culture test.
- Chest CT scan for infection site identification.
- Serologic tests for atypical pneumonia and urinary antigen tests for Legionella and Pneumococcal antigens.
- PCR viral respiratory panel.
- Abdominal ultrasonography, CT scan, and MRI based on clinical suspicion.

#### 9.5.2 Additional tests based on clinical suspicion

- Galactomannan serum antigen and 1-3-β-D-glucan for suspected fungal infection.
- Search for herpetic viruses, mycobacteria, and cryptococcus based on clinical suspicion.

#### 9.5.3 Targeted examinations if initial investigations are inconclusive

- Bronchoalveolar lavage for culture test.
- Histopathological examination of tissue biopsies for direct identification of infectious pathogens.
- Lumbar Puncture and Central Nervous System MRI for neurological signs/symptoms (**Table 5**).

**Table 5:** Infectious workup during ibrutinib therapy.

	Test
Clinical Presentation Assessment:	Blood cell count, chemistry, C-reactive protein, and procalcitonin
	Blood and urine cultures, sputum culture test
	Chest CT
	Serologic tests for atypical pneumonia, antigen tests for Legionella and Pneumococcal
	PCR viral respiratory panel
	Abdominal ultrasonography, CT scan, and MRI based on clinical suspicion
Additional Tests Based on Clinical Suspicion	Galactomannan serum antigen and 1-3-β-D-glucan
	herpetic viruses
	mycobacteria
	cryptococcus
Targeted Examinations if Initial Investigations are Inconclusive	Bronchoalveolar lavage for culture test
	Histopathological examination of tissue biopsies
	Lumbar Puncture and Central Nervous System MRI

### 9.10 Temporary interruption of ibrutinib during infectious events

1. Recommendations suggest *temporarily interrupt* ibrutinib in hospitalized patients until a diagnosis is made and improvement occurs.
2. Resumption of ibrutinib after infectious events varies based on the nature of the infection, with considerations for fungal infections and drug interactions.
3. Outpatient management of fever, if controlled within 72 hours, may allow the continuation of ibrutinib (**Table 6**).

**Table 6:** Temporary interruption of ibrutinib during infectious events.

	*Temporary interruption*	
	YES	NO
New-onset fever and hospitalization due to fever	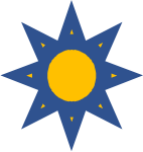	
Suspected or confirmed IFI	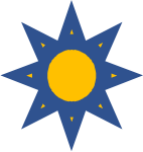	
New-onset fever at home		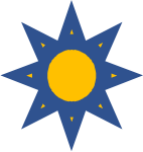
Fever or signs/symptoms of infection worsen after 72 hours	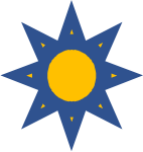	

### 9.11 SARS-COV-2 recommendations

1. Patients with CLL face an increased risk of poor outcome with COVID-19, influenced by age, comorbidities, and impaired immunity.
2. Vaccination is crucial for prevention, even though patients with CLL exhibit variable responses, emphasizing the importance of booster shots.
3. Antiviral agents should be administered, and prophylactic monoclonal antibody injections might be considered based on the circulating variant.
4. Continuous vigilance, testing, and prompt medical attention in case of suspected or active infection is crucial (**Table 7**).

**Table 7:** SARS-COV-2 Recommendations.

	Recommended	
	YES	NO
Vaccination	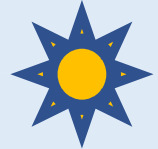	
Antiviral agents	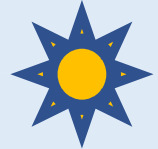	
Prophylactic monoclonal antibody	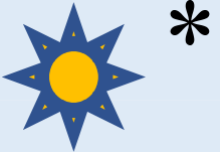	
Monoclonal antibody	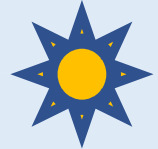	
Continuous vigilance, testing, and prompt medical attention	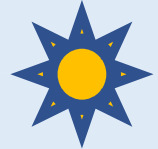	

*might be considered based on the circulating variant.

## 10 Conclusions

The proactive approaches, monitoring, and control of infectious complications during ibrutinib treatment in CLL is a challenging and somewhat controversial topic. Currently, specific guidelines are lacking, but numerous clinical evidences have been derived from prospective and retrospective studies. In a recent study, the latest and current guidelines were analyzed and revised, revealing slight differences in recommendations, likely attributed to the poor quality of data and the heterogeneity of patients included in observational studies (93). This paper is the outcome of extensive and prolonged discussions among a group of peer hematologists aiming to reach a common synthesis in the management of this crucial topic. A work that can be widely shared and prove beneficial in everyday clinical practice.

The management of infectious risks during ibrutinib therapy involves a nuanced approach, balancing the potential complications with appropriate prophylaxis measures. Close collaboration between hematologists and infectious disease specialists is vital to tailor strategies based on individual patient factors, treatment history, and the evolving understanding of infectious risks associated with Ibrutinib in patients with CLL.

In conclusion, managing infections in patients with CLL demands an approach that encompasses a profound comprehension of the disease, treatment modalities, and the ever-changing terrain of infectious risks, especially in the context of emerging pathogens such as SARS-CoV-2. A holistic strategy involves integrating vaccination, adjusting treatments as needed, and implementing proactive measures to counteract infectious threats, all aimed at optimizing outcomes for patients with CLL. As ongoing research and clinical experiences progress, these guidelines will evolve to better align with the dynamic interplay of infectious diseases and therapeutic advancements.
